# Sex and gender bias in the experimental neurosciences: the case of the maternal immune activation model

**DOI:** 10.1038/s41398-019-0423-8

**Published:** 2019-02-14

**Authors:** Pierluca Coiro, Daniela D. Pollak

**Affiliations:** Department of Neurophysiology and Neuropharmacology, Center for Physiology and Pharmacology, Medical University of Vienna, Vienna, Austria

## Abstract

Recent and rapidly developing movements relating to the increasing awareness and reports of gender bias, discrimination, and abuse have reached the academic environments. The consideration that negative attitudes toward women and abuse of power creates a hostile environment for female scientists, facilitating sexual harassment and driving women out of science, can be easily related to. Rationally inaccessible gender biases are not only evident at the level of the researchers, but are also paralleled by a corresponding imbalance at the level of the research subjects. Here, we focus on the maternal immune activation (MIA) animal model to illustrate exemplarily the current state of ex-/inclusion of female research subjects and the consideration of sex as biological variable in the basic neurosciences. We demonstrate a strong sex disparity with a major emphasis on male animals in studies examining behavioral and neurochemical alterations in MIA offspring. We put forward the hypothesis that this neglect of female subjects in basic research may stem from a hard-wired sex/gender bias, which may also be reflected in a similar attitude toward female scientists. We suggest exploring the possibility that by dismantling sex bias and male dominance in basic research one would get an additional handle on favorably modifying the perception and appreciation for women in science.

## Introduction

Despite decade-long efforts, including officially declared intentions and a variety of initiatives for enhancing gender equality, gender biases in scientific environments still exist today and powerfully impact diverse facets of research and academic life and career. The dramatic underrepresentation of female scientists in positions of power and status, in academic and private institutions, is mirroring the gender mismatch in authorships on scientific papers^1^ with female authors appearing much less frequently as first or last author in high-impact journals^2^. The publishing situation itself may be influenced by gender biases during the review process^3^. In addition, subtle and unconscious acts of discrimination during the recruitment process^4,5^ are also in play, preventing women from becoming laboratory heads and reaching positions, which would naturally allow them to feature as the prominent “last” or “senior” author on scientific publications.

Over the past months the rapidly developing #Metoo movement has reached the academic environments and several prominent cases have unfolded since^6–9^. It has been concluded that in academia cases of sexual harassment are more frequent than in most other areas of public life^10^. Consequently, several scientific societies and funding agencies have published declarations concerning their policies toward sexual harassment and issued statements regarding ensuing consequences within the framework of the respective infrastructures. The consideration that negative attitudes toward women and abuse of authority and power creates a hostile environment for female scientists, on the one hand facilitating sexual harassment and abuse, on the other hand driving women out of science can be easily related to.

Facing this situation in 2019 begs the question why even in science, where the ability to most objectively and rationally evaluate data is a core principle and prerequisite for true achievements and progress, such dramatic biases, nurtured by emotionality and subjective believes, persist so tenaciously. Why are some of the people who describe themselves as being fair and objective^11^ still so vulnerable to making biased decisions when it comes to the evaluation of female scientists and scientific results produced and/or presented by a woman? The answer can be found at the very core of the issue itself: Gender bias, like other forms of prejudices and unintentional discrimination, is not subject to conscious reflection and decision making but rather determined by automated emotional processes, subtly bypassing rational and critical internal reviewing structures^12–14^.

These deeply rooted and rationally inaccessible biases are not only evident at the level of the researchers but penetrate into and are paralleled by a corresponding imbalance at the level of the research subjects. Sex and gender differences in drug metabolism, reactions, and side effects are well-described, generally accepted and have led to official requirements to integrate men and women in all study populations comprising human subjects (NOT-OD-15-102, National Institutes of Health, 2015 https://grants.nih.gov/grants/guide/notice-files/NOT-OD-15-102.html). Basic research, however, is lagging behind, as it is still heavily concentrated on male model animals, largely ignoring and/ or not addressing sex as possible variable in scientific data sets. It is more than obvious that preclinical work, which may eventually inform translational research and clinical research questions and therapeutic developments, needs to strive for the inclusion of aspects of sex and sex-dependent effects and functions if both women and men are considered as final beneficiaries of biomedical research.

The misrepresentation of female subjects in basic research can be “historically” explained, in part founded on incorrect perceptions, such as that experiments conducted in female animals are inherently more variable than those relying on males^15^. One may argue that this sex mismatch additionally constitutes the reflection of the biased perception that males constitute “the norm” and relevant “standard population”. Notably, results obtained in male animals are rarely being questioned for their generalizability, whereas research conducted in females is more often than not coined “sex-specific” or “gender-relevant” with the extent to which it can be applied to the general (male) population remaining to be further determined.

To date, most preclinical biomedical research has been conducted with inadequate consideration of the sex of the experimental subjects studied^16–18^. In the current article, we are focusing on the experimental animal maternal immune activation (MIA) model to exemplarily illustrate the current state of sex-/inclusion of female research subjects and the consideration of sex as biological variable in basic (neuroscience) research. We summarize, integrate, and discuss MIA literature for the time period of 2000–2018. Focusing on three levels of phenotypic characterization of MIA offspring (behavior, neurotransmitter, and cytokines analysis), we demonstrate a strong sex disparity with a major emphasis on male animals in the available studies and reflect on consequences and future perspectives.

## Overview of the MIA model

The MIA model of neuropsychiatric disorders was founded based upon a series of epidemiological studies, which had demonstrated an association between maternal infection during pregnancy and offspring autism spectrum disorder or schizophrenia^19–26^ diagnoses. Recently, the range of psychiatric disturbances associated with gestational infections has been extended to include an increased risk for offspring bipolar disorder, major depression, epilepsy, and cerebral palsy^20,27,28^. It is considered that rather than damage being induced by the infectious agent itself, maternal infection disrupts the delicate immune balance between the maternal and fetal environments. Hereby, the milieu for the developing fetal nervous system is altered paving the way for the occurrence of aberrant brain structures and functions, which form the basis for an augmented risk for offspring mental disorders^20,23^. Several animal models of MIA based upon the administration of immunogenic substances to the pregnant female have been developed in search for the pathophysiological mechanisms underlying the detrimental effects of gestational infection.

Pioneering studies were designed based upon the reported association between prenatal influenza infection and a diagnosis with schizophrenia in the adult life^29^ and used live human influenza virus to induce MIA in mice^30^. Although directly employing a pathogenic virus has the advantage that a full spectrum of naturally occurring immune response can be elicited, the field has since moved toward mainly using Poly (I:C) (polyinosinic-polycytidylic acid) and LPS (lipopolysaccharide) to induce MIA in laboratory animals. These substances, which mimic viral and bacterial infection, respectively^27^ induce a limited, but well-defined immune response allowing for a precise control over the intensity and timing of MIA as critical determinants for offspring phenotype^31–33^. In addition, variations in other experimental parameters including the experimental animal’s strain and age, housing conditions, and other environmental variables contribute to the heterogeneity of MIA paradigms and the effects observed^32,34–36^. For the sake of enhancing transparency and reproducibility and to minimize bias in the MIA mouse model, the request to standardize and meticulously report on the applied MIA model has been recently been put forward, including the integration of major experimental variables at the side of the offspring^37^. Here, the age at which the animals are being examined, the brain region analyzed and, particularly, offspring sex is considered as significant modulatory impact. Sex-dependent time points of vulnerability and resilience and sex-specific responses to prenatal stress are known to shape an individual’s response to stress over the lifetime and determine the risk for the development of mental illness^20,22^. These examples illustrate a general principle, which demands the inclusion of sex as a biological variable (SABV) in biomedical research as prerequisite for improving our understanding of disease mechanisms and the development of preventive and therapeutic approaches.

## Sex bias of experimental animals in MIA studies—overview

For the present analysis studies reporting behavioral, cytokine, and neurotransmitter offspring phenotypes in MIA animal models, were collected by literature search (key words have: “maternal immune activation AND behaviors”; “maternal immune activation AND cytokines”; “maternal immune activation AND neurotransmitter”; “prenatal exposure to infection”; “gestational inflammation”; “gestational infection”; “gestational immune activation”) using PubMed (*n* = 733) and ScienceDirect (*n* = 676) limited to the time period between 01 January 2000 to 01 September 2018. After removal of duplicates, the total number of records was *n* = 1003. Eight hundred and twenty-eight manuscripts (after exclusions of review articles, conference abstracts, editorials and letter of comments, book chapters, and not English articles (*n* = 175)) were screened and included in the subsequent analysis. Of these articles, only manuscripts using mammals as experimental animals and MIA induction based upon LPS and/or Poly (I:C) injection, IL-6, *Escherichia coli*, stress-induced stimulation or influenza virus infection, *n* = 432 were considered. Studies focused only on the pregnant mothers, placental studies only, preterm labor, effect of infection on abortion rates/fetus absorption, mother treated with ethanol and/or high/low-fat diet, trial studies on human (women and/or child) were not included (*n* = 396). The PRISMA flow diagram was used in order to summarize the search and study selection processes (Supplemental Fig. 1).

**Fig. 1:**
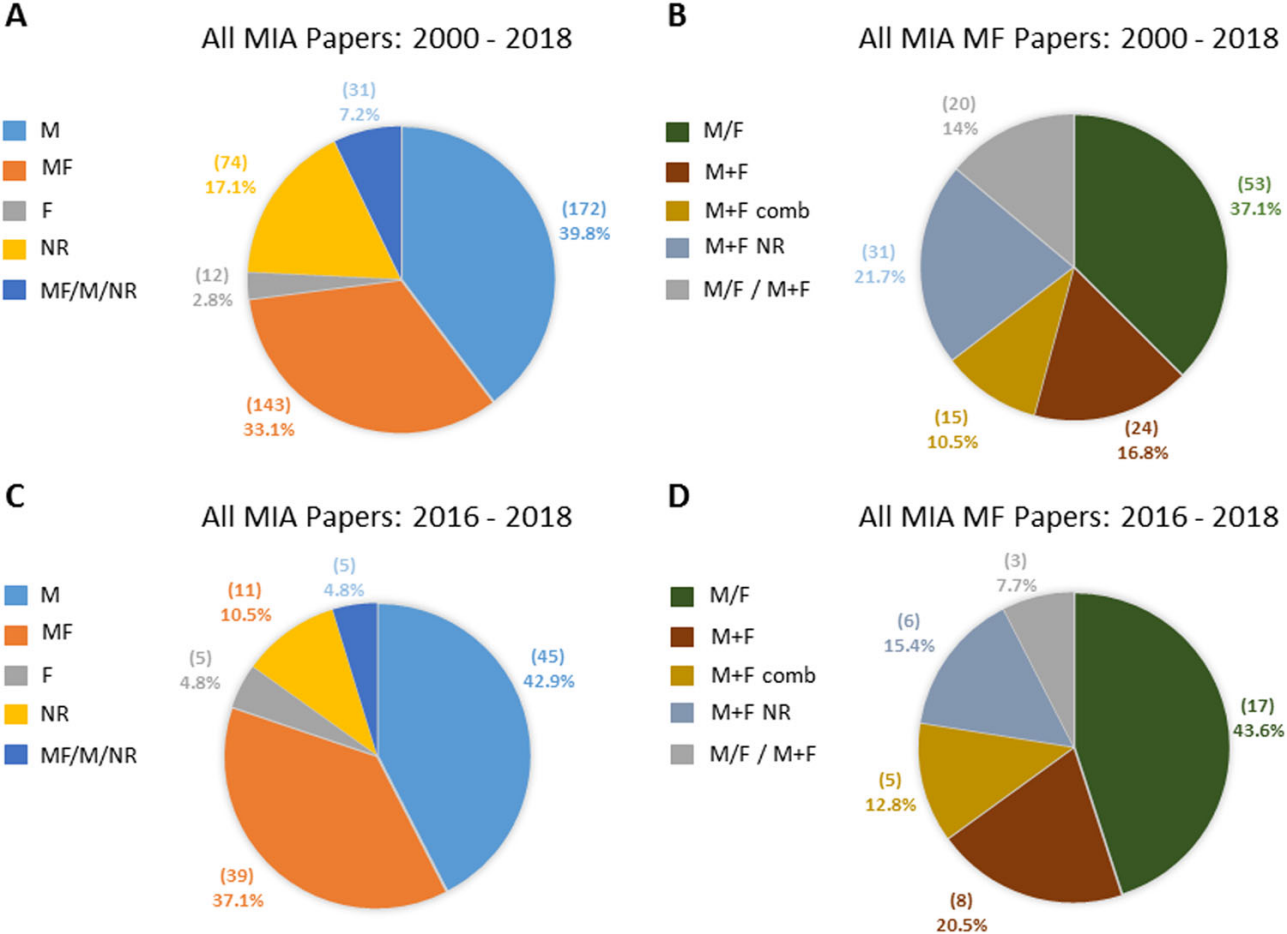
Distribution of offspring sex analyzed in mammalian maternal immune activation studies published 2000–2018 and 2016–2018. **a** Offspring sex represented in data of 432 MIA papers analyzed in the present article between 2000 and 2018. **b** Categorization of articles that have used analyses in both male and female MIA offspring. **c** Offspring sex represented in data of 105 MIA papers analyzed in the present article between 2016 and 2018. **d** Categorization of articles that have used analyses in both male and female MIA offspring related to the 2016–2018 time period. Actual numbers of identified studies and relative percentages are displayed in the pie chart. M = male offspring only; MF = male and female offspring; F = female offspring only; NR = not reported; MF/M/NR = combination of male and female offspring and/or male only and/or not reported. M/F = male and female analyzed separately; M + F = male and female combined with analysis by sex; M + F comb = reported no sex effects but did not show the respective analysis and combined male and female data; M + F NR = both male and female combined for all the experiments without reporting whether or not sex effects had been analyzed; M/F, M + F, M + F comb, M + F NR = combination of M/F and/or M + F, M + F comb, M + F NR (at least in one set of experiments)

Of the herby identified articles (Supplemental Table 1) ~ 40% used male offspring only (M), whereas < 3% relied exclusively on female offspring (F). About one-third of the included articles used both male and female offspring (MF). The remaining 20% of studies was based on various combinations of the use of males and females in different experiments (MF/M, 7.2%) or did not specify the sex of the offspring (NR, 17.1%) or a combination thereof (Fig. 1a).

However, not all studies that used both males and females indeed analyzed the data by sex. To better understand this point we classified MF papers into four categories: (i) analysis of male and female data separately (M/F); (ii) inclusion of male and female data with an analysis by sex (M + F); (iii) merging of male and female data indicating that no sex effects had been found without showing the respective analysis (M + F comb) and (iv) studies using both male and female subjects without indicating whether an analysis by sex had been conducted (M + F NR). We found that the MF group is composed of 37.1% of M/F, ~ 17% of M + F and a 14% studies with both M/F and M + F analysis, followed by a 10% of M + F comb and 21.7% of M + F NR (Fig. 1b). Of note, 32% of total MF studies, did not present any sex-analysis. Already in 2001, a statement released by the NIH clearly demanded that “if information about the existence of sex differences is absent or equivocal then both sexes should be studied in numbers sufficient to permit valid analysis” (https://grants.nih.gov/grants/guide/notice-files/NOT-OD-02-001.html). Effective 25 January 2016, the NIH implemented a policy aimed at integrating sex and gender science as an integral component of methodological rigor and reporting in health research (NOT-OD-15-005 https://grants.nih.gov/grants/guide/notice-files/not-od-15-005.html), which expects scientists to account for the possible role of SABV in vertebrate animal and human studies. Moreover, on 9 June 2018, the Human Subjects System replaced the Inclusion Management System for reporting participant sex/gender, race, and ethnicity information (NOT-OD-18-179 https://grants.nih.gov/grants/guide/notice-files/NOT-OD-18-179.html). Nonetheless, single sex studies of males still largely predominate in the MIA literature during the 2016–2018 period increasing to ~ 43% (Fig. 1c), whereas MF articles constitute ~ 30% of MIA papers without any sex-analysis. At present, <3 years after implementation of the NIH SABV policy, this preliminary analysis can be considered a trend at best and in ~ 5 years' time a re-evaluation of published articles should be better suited to determine the practical impact of this policy on the MIA field, as it is likely that that manuscripts published in 2016–2018 reflect studies that were designed and/or funded prior to the release of NOT-OD-18-179.

In order to examine whether there could be a tentative association between the gender of the senior (lead) author and the sex of the experimental animal in the MIA studies, authors' gender on all studies included in the present analysis was inferred based upon first name, published photos and other text references as previously described^38^. As depicted in Supplemental Fig. 2 the only male MIA studies (M) were predominantly published by a male group leader (76.4% versus 23.6%), the studies that used both male and female MIA offspring (M/F) showed a slight increase in the percentage of female group leaders (38%), whereas for the studies using only female MIA offspring (F) the gender distribution pertaining to the last authorship was comparable to the M group (however the number of F studies included was much lower than for the other two groups, *n* = 12). However, given the overall small number of manuscripts included herein it is important to unmistakably emphasize that this first glance on the potential relevance of author gender for the choice of sex of the experimental subjects used, is not meant to and cannot provide any definitive answer on this matter. Larger-scale, specifically designed studies with different and dedicated analytical instruments will be needed in order to properly address this question.

## Sex disparity in behavioral studies of MIA offspring

Offspring of mothers experiencing gestational immune activation have been repeatedly demonstrated to feature behavioral impairments related to several neuropsychiatric disorders. The behavioral phenotypes revealed include abnormal social behaviors, repetitive behaviors, depression-like, and anxiety-like behaviors as well as memory deficits^34,36,39–46^. The tradition of performing behavioral experiments mainly in male rodents can be related to the early discovery of estrous-linked changes in locomotor activity in rats^47^, which alerted experimenters to hormonally programmed changes in females as possible confounds of non-reproductive traits. However, few systematic studies have directly compared the variability in behavioral studies conducted in male versus female rodents. A meta-analysis of 40 inbred strains of mice concluded that the assumption that female mice exhibit more variable response than males is not substantiated by experimental evidence^15^. A recent review supported this notion stating that “the belief that non-human female mammals are intrinsically more variable than males and too troublesome for routine inclusion in research protocols is without foundation” and further reported a particular sex bias in neuroscience research where studies relying exclusively on male animals surpassed those using females 5.5–1^48,49^.

**Fig. 2:**
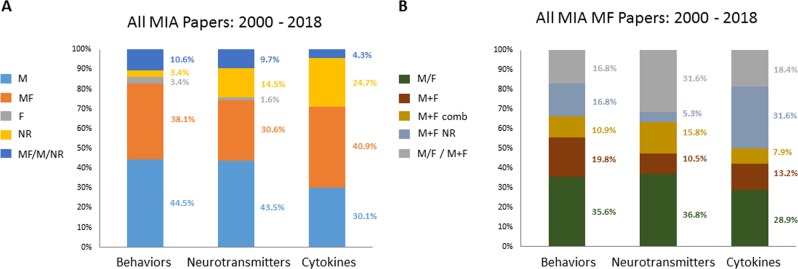
Distribution of sex in studies examining behavioral and neurochemical offspring phenotypes in mammalian maternal immune activation studies published 2000–2018. **a** Offspring sex represented in data of MIA papers examining behavioral, neurochemical, and cytokine offspring phenotypes analyzed in the present article. **b** Categorization of articles that have used both male and female MIA offspring in at least one set of experiments presented. Actual percentages are displayed in the chart. M = male offspring only; MF = male and female offspring; F = female offspring only; NR = not reported; MF/M/NR = combination of male and female offspring and/or male only and/or not reported. M/F = male and female analyzed separately; M + F = male and female combined with analysis by sex; M + F comb = reported no sex effects but did not show the respective analysis and combined male and female data; M + F NR = both male and female combined for all the experiments without reporting whether or not sex effects had been analyzed; M/F, M + F, M + F comb, M + F NR = combination of M/F and/or M + F, M + F comb, M + F NR (at least in one set of experiments)

Indeed, the influence of sex on brain function health and disease is underexplored in neuroscience research^47,49–51^ albeit apparent gender-dependent distribution patterns in the prevalence of several psychiatric disorders, including those whose symptoms are being modeled in MIA offspring (e.g., schizophrenia, autism spectrum disorder, attention deficit disorder, mood, and anxiety disorders^52–54^. However, astoninglishly little emphasis has been placed on the examination of possible sex effects and sex × treatment interactions in MIA offspring with respect to the analysis of the behavioral phenotypes relevant to the above-mentioned mental illnesses. The greatest part of studies constituted articles containing behavioral data on male offspring only (44.5%), whereas only 3.4% had conducted studies solely in female MIA offspring. Of the ~ 38.1% of MF studies, the M/F group comprised 35.6%, the M + F group ~ 20% and the combination of both ~ 17%, mirroring the overall trend (Fig. 2a, b). However, properly conducted studies directly comparing phenotypes in male and female offspring can yield valuable insights as exemplarily demonstrated in some of the studies included in the present analysis. e.g., Arad et al.^55^ demonstrated that male offspring of Poly(I:C) injected mothers but not their female littermates, exhibited abnormally persistent latent inhibition and slower reversal compared with controls. Reversely, only female offspring exhibited increased immobility and decreased saccharine preference in the forced swimming test and saccharine preference test. Interestingly, employing a different MIA protocol anxiety- and depression-related behavior were also exclusively observed in adult male offspring^56^. On the other hand, no effect of gestational Poly(I:C) challenge on prepulse inhibition (PPI) was found in male rat offspring exclusively in one study^57^, whereas others reported no effect in either sex^58^. Interestingly, in the LPS MIA model a PPI deficit was observed in male offspring only^31^.

## Sex bias in neurochemical analyses in MIA offspring

MIA offspring exhibit neurochemical changes that are characteristic of several neuropsychiatric disorders^27–29,59^. Serotonin and dopaminergic signaling is altered in offspring across different MIA models^27–29^. In addition, specific changes in inhibitory neurotransmission have been linked to both schizophrenia and autism spectrum disorders^60^ and similar reductions in several components of the GABAergic system have been repeatedly identified in the brain of MIA offspring^27–29,59,61,62^.

Here we found that despite well-established sex differences in sex hormones and their interactions with neurotransmitter systems (e.g., review^63^) > 43% of MIA studies examining neurotransmitter changes focused on male offspring only and 30% were MF studies. Of these, 37% were M/F and 10% only M + F studies, including a direct statistical analysis of SABV (Fig. 2a, b). Related to the neurotransmitter analysis and at the core of the MIA model, is the analysis of pro-inflammatory cytokines and inflammatory mediators^34,64,65^, which may pass from the maternal to the fetal compartment hereby disrupting normal brain development^66–68^ and compromising neuronal excitability and neurotransmitter function^69^. Individual studies have observed sex-dependent changes in central nervous system outcome owing to prenatal infection, indicating that offspring sex may be relevant as factor contributing to outcome variability (e.g., ref. ^70–72^).

Regarding the consideration of sex effects in the 93 MIA publications that analyzed offspring cytokine levels, we observed a slightly higher percentage of MF and a lower percentage of male only studies, in comparison with those articles focusing on behavioral and neurotransmitter assessment. No studies had been conducted using only female offspring (Fig. 2a) but almost the 40% of the MF studies have not provided sex-based analysis (M + F comb and M + F NR (Fig. 2b). However, important insights regarding sex-specific outcomes of MIA face to cytokine levels and neurotransmitter functions can be obtained when a proper experimental design is employed. For example, in MIA challenged Long-Evans rats female offspring rats had higher increases in the levels of IL-6 mRNA in the hippocampus than males and only males presented with impaired spatial learning^73^.

## Conclusion and perspective

An animal model in preclinical research is neither expected to have lockstep relationships with outcomes in people, nor to be an exact replication of the differences found in the human population^74^. However, its powerful potential lies precisely in the possibility to examine, under controlled conditions, individual variables potentially impacting on, or modulating human health and the course of disease.

This potential remains underexplored with regards to the examination of sex differences in the MIA model as we here reveal a substantial sex disparity in offspring analysis in publications over the last 18 years. We find that in a large majority of articles the effect of MIA has been exclusively analyzed in male offspring, whereas the percentage of articles employing female subjects is represented in the single digits, defying the argument that only one sex had been “randomly” analyzed^75^.

The arguments for why female subjects need to be included in biomedical research, starting from basic science study designs, have been eloquently and comprehensively delineated elsewhere^76–81^. The evidence that the integration of data of both sexes is important and necessary in preclinical research for completing our understanding about disease mechanisms as well as for driving the path to novel and advanced diagnostic and therapeutic strategies is logically sound and pragmatically compelling.

Even more, for models of illnesses for which epidemiological studies clearly point toward sex-specific prevalence and manifestation, including several neuropsychiatric disorders, which can be studied using the MIA model^28,82^, the analysis of female and male subject appears as a fundamental prerequisite. Undoubtedly, gender effects in the human population are not exclusively mediated by biological mechanisms, but rather result from complex socio-cultural influences interacting with the endogenous, internal foundations of the individual. The power of animal research lies in the possibility to employ a reductionist approach to single out individual of those mechanisms in a prospective study design to complement existing and inform future human studies.

Despite a wealth of animal research on the effects of MIA on brain development, function, and behavior, processes that are known to be modulated by sex effects^63,83–85^, the interplay between the factor “treatment” and “sex” remains poorly explored in MIA research as summarized here. This overview can therefore be viewed as example for the basic and translational neurosciences, where overall, the integration of female and male subjects has systematically failed up to today.

The obvious question is therefore: why? Why is it that in a time, in which we are aiming to tailor personalized medicine approaches to individual, more or less frequent, variants of selected genetic loci, the largest, chromosomically determined factor allowing to differentiate ~ 50% of the population, i.e., the factor “sex”, is being so readily underappreciated?

Does this repeated failure to integrate female together with male subjects in basic biomedical research and to examine—in proper statistical terms—potential sex differences represent a conscious or unconscious intentionality? The easy answers to these questions are apparent and have been delineated before: “historic” developments of paradigms, the mostly refuted argument about larger variability in female cohorts of experimental animals (which, even if true, would not suffice as an argument not to study a “biological condition” applying to half of the population), the increase in workload and costs that comes with doubling animal number by including both sexes etc.

However, what if this sex bias in basic research stems from a more deeply rooted, hard-wired, consciously inaccessible, and logically uncontrollable neglect of the relevance of female research subjects? What if this sex bias was also reflected in a similar attitude toward female scientists and women in society in general? This hypothesis can neither be experimentally falsified nor verified to date. However, do the lean numbers of women in leadership positions (in science) and their modest increases despite great efforts, together with the tip of the iceberg of sexual misconduct and assault revealed by the #Metoo movement, not suffice to explore the possibility that by dismantling sex bias and male dominance in basic research we would get an additional handle on favorably modifying the perception and appreciation for women in society? Would equity not provide a short-cut to excellence, if the full potential of ~ 50% of people could add an additional/ different source of intellectual insight and execution of leadership tasks?

How long would it take to observe measurable changes?

Evolutionary biology holds an answer to this question: the rate of change is largely dependent on the environmental pressure.

## Supplementary information

Supplemental Figure 1

Supplemental Figure 2

Supplemental Figure Legends

Supplemental Table 1

Supplemental Table 2

Supplemental Table 3
