# Supplementary figures and images for: Sex and gender bias in the experimental neurosciences: the case of the maternal immune activation model
Source: Transl Psychiatry. 2019 Feb 14;9:90. doi: 10.1038/s41398-019-0423-8 (PMC6375995; doi:10.1038/s41398-019-0423-8)

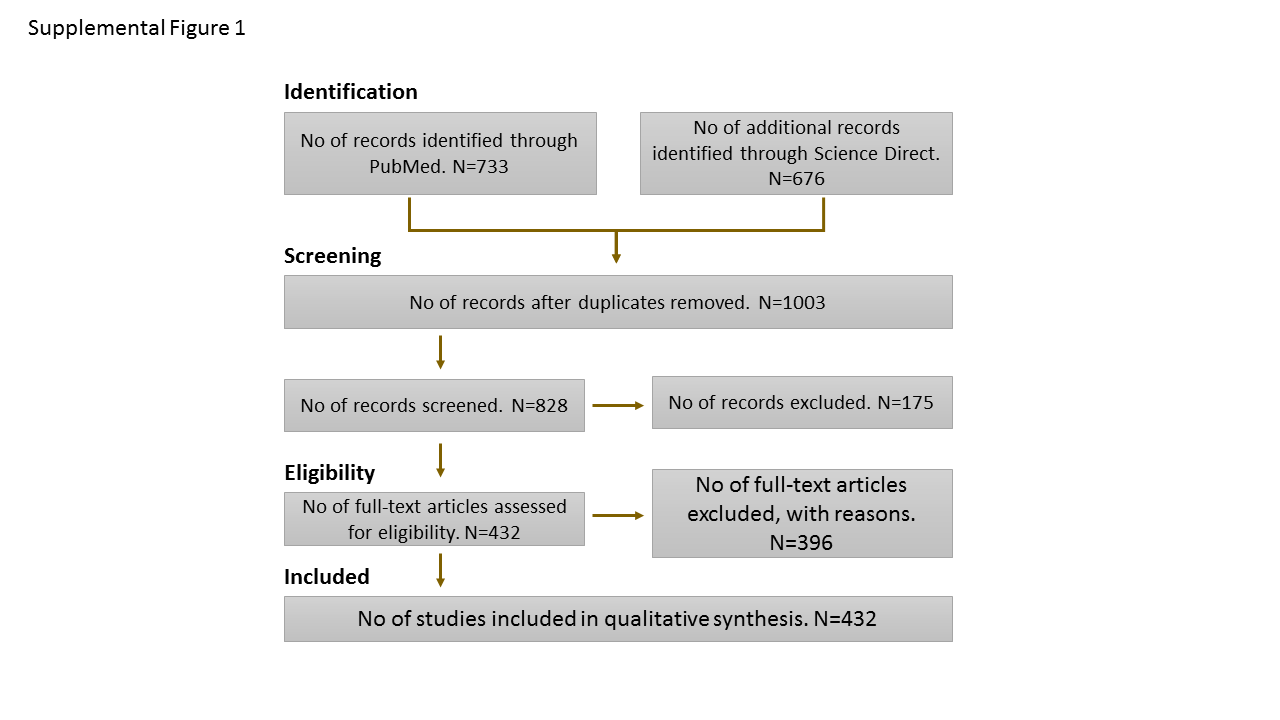

Supplement: Supplementary file 1 — Supplemental Figure 1 [file 41398_2019_423_MOESM1_ESM.tif]

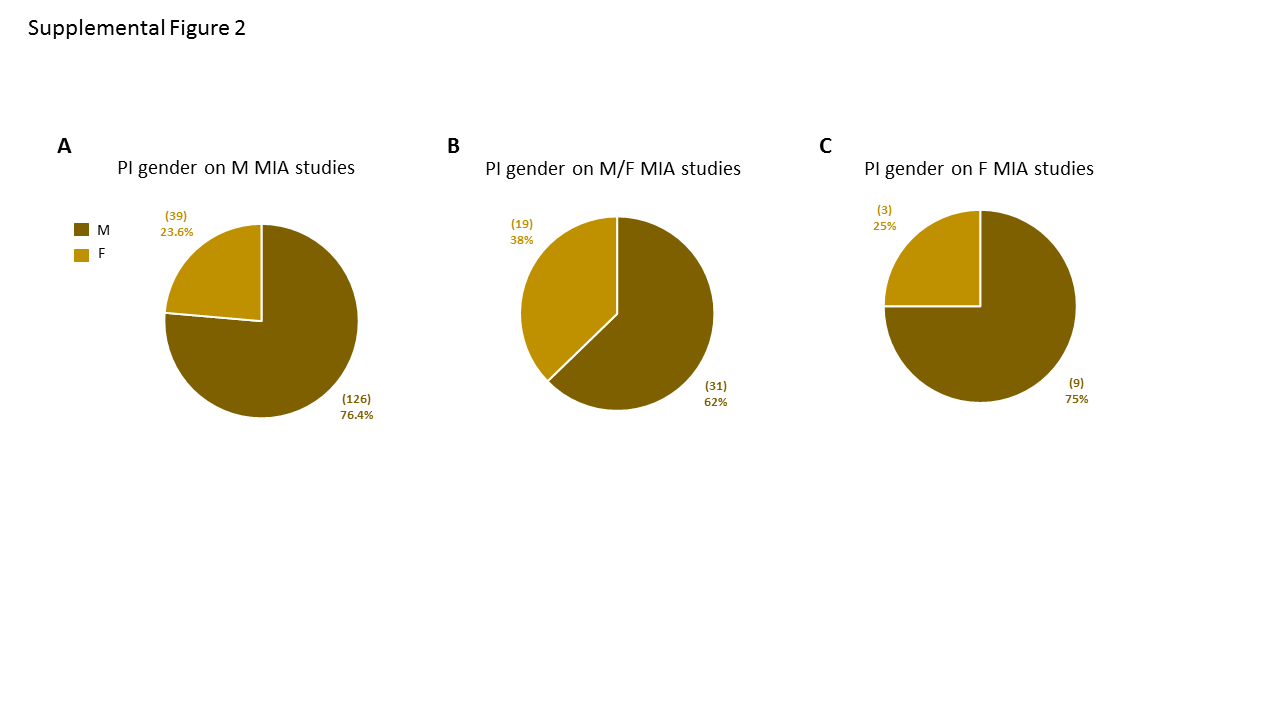

Supplement: Supplementary file 2 — Supplemental Figure 2 [file 41398_2019_423_MOESM2_ESM.tif]
