# Supplementary material for: Sex and gender bias in the experimental neurosciences: the case of the maternal immune activation model
Source: Transl Psychiatry. 2019 Feb 14;9:90. doi: 10.1038/s41398-019-0423-8 (PMC6375995; doi:10.1038/s41398-019-0423-8)
Supplement: Supplementary file 3 — Supplemental Figure Legends [file 41398_2019_423_MOESM3_ESM.docx]

**Supplemental Figure 1. Distribution of the gender of the senior author and the sex of the experimental animal in the MIA studies**

**A.** Senior author gender on male offspring only MIA studies (M), (**B**) male and female MIA studies (M/F) and (**C**) female offspring only. Actual percentages are displayed in the chart.

**M** = male author; **F** = female author.

**Supplemental Figure 2. PRISMA flow diagram Chart**

Flow of information through the different phases of this systematic review.
